# Validation of a synthetic lumbar spinal endoscopy simulator: skills transfer to real surgery

**DOI:** 10.1590/0100-6991e-20260022-en

**Published:** 2026-02-15

**Authors:** ÁLYNSON LAROCCA KULCHESKI, PAUL ANDRÉ ALAIN MILCENT, XAVIER SOLER I GRAELLS, JOÃO GABRIEL BELEGANTE SCALABRIN, CAROLLINE POPOVICZ NUNES, EDMAR STIEVEN

**Affiliations:** 1 - Universidade Federal do Paraná (UFPR) - Departamento de Cirurgia, Ortopedia e Traumatologia - Curitiba - PR - Brasil

**Keywords:** Endoscopy, Medical Education, Medical Students, Simulation Training, Spine, Validation Study, Educação de Graduação em Medicina, Endoscopia, Treinamento por Simulação, Coluna Vertebral, Estudantes de Medicina, Estudo de Validação

## Abstract

**Introduction:**

Surgical simulators offer substantial benefits for technical skills training by providing a risk-free environment for practice. However, access to effective simulators in surgical education remains limited. This study aimed to validate a synthetic lumbar spinal endoscopy simulator through skills transfer and assess its educational applicability.

**Methods:**

Forty medical students were randomized to simulator training (n=20) or control (n=20). After training, all performed supervised diagnostic endoscopy. Procedures were recorded and evaluated by a blinded examiner for total time, look-downs, instrument loss, supervisor interventions, and Global Operative Assessment of Laparoscopic Skills (GOALS). A Likert-scale questionnaire assessed perceptions of simulation training.

**Results:**

The intervention group showed superior performance, with reductions of 43.7% in procedure time, 85.3% in look-downs, 75.9% in interventions, 93.3% in instrument loss duration, and 91.2% in total loss percentage (p<0.001). GOALS scores were significantly higher in all domains (p<0.001). All participants endorsed incorporating simulation into medical education.

**Conclusion:**

The simulator demonstrated strong transfer validity, significantly improving surgical performance. GOALS scores tripled among simulator-trained participants, and acceptance of the simulator for educational use was unanimous..

## RESUMO

**Introdução:**

Os simuladores cirúrgicos oferecem benefícios substanciais para o treinamento de habilidades técnicas, proporcionando um ambiente sem riscos para a prática. No entanto, o acesso a simuladores eficazes na educação cirúrgica ainda é limitado. Este estudo teve como objetivo validar um simulador sintético de endoscopia lombar por meio da transferência de habilidades e avaliar sua aplicabilidade educacional.

**Métodos:**

Quarenta estudantes de medicina foram randomizados para treinamento no simulador (n=20) ou grupo controle (n=20). Após o treinamento, todos realizaram endoscopia diagnóstica supervisionada. Os procedimentos foram gravados e avaliados por um examinador cego quanto ao tempo total, desvios de olhar para baixo (look-downs), perda de instrumento, intervenções do supervisor e escore Global Operative Assessment of Laparoscopic Skills (GOALS). Um questionário em escala de Likert avaliou percepções sobre o treinamento em simulação.

**Resultados:**

O grupo intervenção apresentou desempenho superior, com reduções de 43,7% no tempo de procedimento, 85,3% em look-downs, 75,9% em intervenções, 93,3% na duração da perda de instrumentos e 91,2% no percentual do tempo total em perda (p<0,001). Os escores GOALS foram significativamente maiores em todos os domínios (p<0,001). Todos os participantes apoiaram a incorporação da simulação na educação médica.

**Conclusão:**

O simulador demonstrou forte validade de transferência, melhorando significativamente o desempenho cirúrgico. Os escores GOALS triplicaram entre os participantes treinados no simulador, e a aceitação para uso educacional foi unânime..

## INTRODUCTION

Spinal endoscopy has emerged in recent decades as an evolution of minimally invasive techniques, offering important advantages over traditional approaches such as reduced anatomical trauma, rapid rehabilitation, and maintenance of spinal stability^1^ ^,^ ^2^. However, its adoption is hindered by technical challenges and a steep learning curve, requiring specific psychomotor skills and mastery of three-dimensional anatomy as viewed on a two-dimensional screen^3^ ^-^ ^5^.

Surgical simulators offer substantial benefits for technical skills training by providing a risk-free environment for practice^5^ ^,^ ^6^. However, access to effective simulators in surgical education remains limited. Cadavers and animal models, although realistic, require specific infrastructure. High-fidelity virtual reality simulators require advanced technology and dedicated software. In contrast, synthetic simulators, especially low-cost options, offer a more viable alternative for medical education institutions, although they have been poorly explored and scarcely validated in the literature^5^.

To ensure the utility of a simulator in medical training, its efficacy must be confirmed through validation processes. Common validation methods involve the comparison of different simulators or the assessment of performance between experienced and inexperienced professionals using the same simulator, allowing for an inference as to whether the simulator contributes to the acquisition of technical skills. However, the most robust form of validation-known as transfer validity-is intended to demonstrate that skills acquired on the simulator translate into improved performance during actual surgical procedures^5^ ^,^ ^7^. This type of study is rare due to complex logistics and ethical requirements^5^ ^,^ ^7^ ^-^ ^9^. To date, no study has demonstrated transfer validity in spinal endoscopy, rendering the present investigation pioneering in the field of minimally invasive surgical education.

Therefore, this study aimed to validate a low-cost synthetic simulator for lumbar spinal endoscopy using the skills transfer method. Additionally, we evaluated the acceptance and applicability of the simulator to medical education.

## METHODS

This randomized educational trial was conducted in both simulated and real-world settings. Approval was granted by the ethics committee of a university hospital (protocol number: CAAE 76671623.1.0000.0096; approval number: 6.667.174). The procedures used in this study adhere to the tenets of the Declaration of Helsinki. Informed consent was obtained from all individual participants included in the study.

Eligible participants were all medical students from the sixth semester onwards who had completed the surgical technique course and possessed no prior experience with lumbar spinal endoscopy. Those who declined to provide written informed consent or who had any previous exposure to the simulator were excluded.

Randomization was performed using the UX Apps Random Number^®^ generator application, and participants were randomly assigned by the principal investigator to one of two groups: intervention (simulator training) or control (no simulator training).

### Simulator

**Figure 1:**
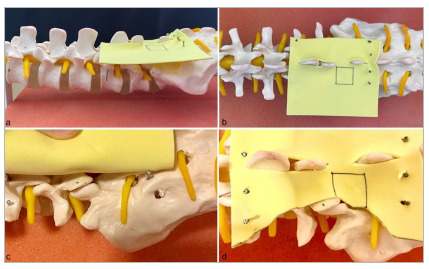
(a) Ligamentum flavum inserted into the spinal model. (b) Square area delineated on the ligamentum flavum. (c) Exposure of the facet joint underneath the ligamentum flavum. (d) Cut through the ligamentum flavum to expose the facet joint between L5 and S1.

**Figure 2:**
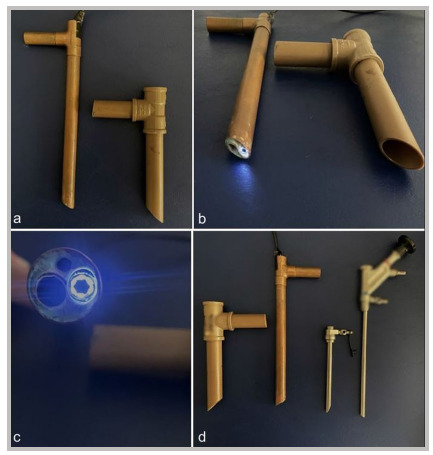
Endoscope light source, comparison with real original model. (a) Overview of simulator set-up: endoscope and working sheath. (b) View of the beveled edge and the 30-degree optical angle, in accordance with the original device. (c) Identification of the light, optical camera and working sheath portals. (d) Comparison between the simulated model and original endoscope.

**Figure 3:**
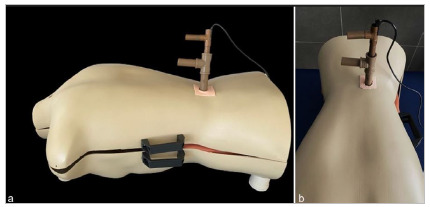
Endoscope inserted into the simulator. (a) Lateral view and (b) anterior view.

This study used a previously developed spinal endoscopy simulator10, which is a low-cost, reproducible, synthetic model with previously established face and construct validity^5^ ^,^ ^10^. The simulator consisted of an opaque plastic manikin containing an adapted L5-S1 spinal segment (model EB-3012, Astral Científica, Curitiba, Brazil), with reproduction of the interlaminar space, ligamentum flavum, nerve root, and intervertebral disc. A single access portal measuring 2.5 × 2.5cm was created in the lumbar region of the manikin. The ligamentum flavum was simulated using yellow ethylene vinyl acetate (EVA) sheets marked with 6.25cm² squares (Figure 1). Endoscopic visualization was achieved using a probe-type camera (model SXT-5.0M, KKMOON, Shenzhen, China) connected via USB to a monitor (Figures 2 and 3). The total cost for the simulator was US$90.0010. The endoscopic scissors and probe used in the simulator were actual instruments, with an approximate unit cost of US$700.00.

### Training protocol

**Figure 4:**
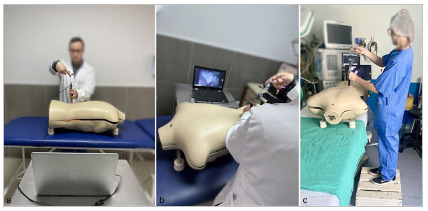
(a, b) Simulator training. (c) Warm-up session.

Participants in the intervention group viewed a ten-minute instructional video and engaged in two practical sessions: an individual simulator-based practice session lasting up to one hour, focused on insertion, triangulation, and identification of anatomical structures; and an additional ten-minute warm-up session on the simulator on the day of the actual surgery, with reinforcement of the triangulation and anatomical identification exercises (Figure 4). The control group received the usual theoretical instruction, including access to the instructional video, without any simulator-based practice.

Simulator training consisted of four sequential steps. Initially, participants familiarized themselves with the simulator and instruments. Subsequently, they introduced the working sheath and the endoscope via the interlaminar approach, with panoramic visualization of the internal structures (ligamentum flavum and facet joint). Once the endoscope was stabilized, the students practiced instrument triangulation. Finally, they performed simulated diagnostic endoscopy, with identification and palpation of two specific targets: (a) a square area delineated on the ligamentum flavum, precisely touching its edges without transgressing them; and (b) the L5 and S1 facet joints. All steps were repeated five times by each participant.

### Real surgery

**Figure 5:**
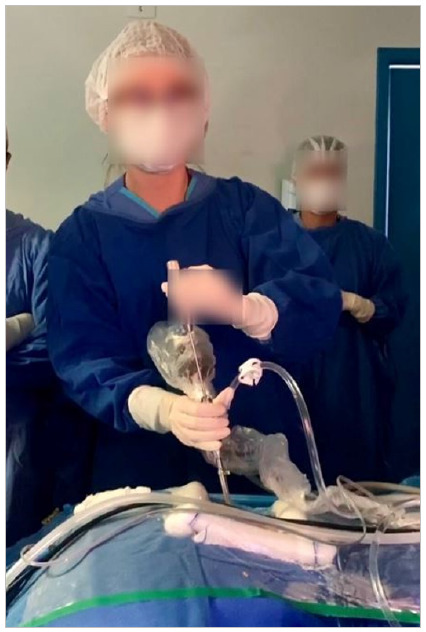
Student performing the activity in real surgical environment..

Two days after training, all students (intervention and control) performed real diagnostic endoscopy under the direct supervision of the attending surgeon, who was blinded to group allocation. The students manipulated the endoscope and probe to identify the ligamentum flavum (four borders) and facet joint (Figure 5). The procedure was recorded for later analysis.

Following the procedure, all participants completed an experience questionnaire using a Likert scale^11^.The questionnaire consisted of four questions, with responses ranging from 1 (strongly disagree) to 5 (strongly agree). The questions were as follows: (1) Do you believe a spine simulator could enhance learning in this course?; (2) Would you like simulator training to be integrated into your undergraduate program?; (3) Do you believe simulator training could replace actual surgical training?; and (4) Do you find the instructional video sufficient preparation for performing the exercise in real surgery?

The recordings were reviewed by an independent examiner who was blinded to group allocation. The following parameters were analyzed: total procedure time; number of look-downs; number of instrument loss episodes (instrument not visible on the display unit); total duration of instrument loss; percentage of total loss duration; number of interventions required by the supervisor; and the Global Operative Assessment of Laparoscopic Skills (GOALS) score, with a maximum score of 25.

To ensure a proportional comparison among all participants, the number of look-downs per minute and the number of instrument loss episodes per minute were also calculated.

### Statistical analysis

Data were analyzed using SPSS, version 20.0. The Shapiro-Wilk test was used to assess the normality of data distribution. Qualitative variables were compared using the McNemar test. The age variable was normally distributed and compared using Student’s t-test for paired samples. For the remaining quantitative variables, which showed a non-parametric distribution, the Wilcoxon test was used for paired comparisons.

To standardize the relative impact of simulator training on participants’ technical performance, the relative percentage difference between each pair of participants in the intervention and control groups was calculated using the following formula: Relative difference (%) = ((value in the intervention group - value in the control group) / value in the control group) × 100. Confidence intervals (95% CI) were reported for these differences. Additionally, effect sizes (r) were calculated, and the cut-off points proposed by Cohen were used to interpret the magnitude of the effects: below 0.30, small effect; from 0.30 to 0.50, medium effect; and above 0.50, large effect.

A post-hoc statistical power analysis was conducted using WINPEPI, version 11.65, to assess the robustness of our results, considering the available sample size and the observed effect size. The significance level was set at 5% for all analyses. 

## RESULTS

The sample consisted of 40 medical students, equally randomized into the intervention (n=20) and control (n=20) groups. There were no statistically significant differences between the groups regarding sex, age, course period, laterality, previous participation in surgical procedures, or interest in pursuing a surgical career. The post-hoc analysis indicated a power of 78.4% to detect moderate differences.

**Figure 6:**
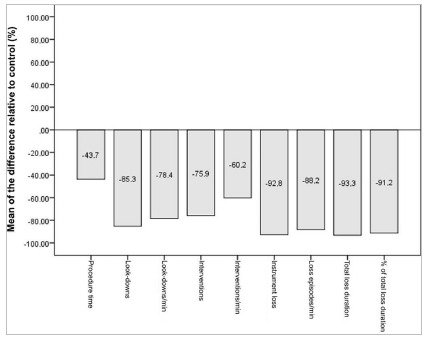
Mean of the percentage difference relative to the control group for technical performance.

Simulator-trained participants showed significantly better results than control participants in all technical performance variables, including shorter total procedure time, fewer look-downs, fewer instrument loss episodes, and fewer interventions by the supervising surgeon (Table 1 and Figure 6).

**Table 1:** Technical performance assessment in the intervention (simulator training) and control (no simulator training) groups.

Performance	Intervention n=20	Control n=20	Mean of the difference relative to control (%) [95% CI]*	p
Procedure time, s	61 (41-114)	123 (78-242)	-43.7% [-51.0 a -36.4]	<0.001
Number of look-downs	0 (0-2)	5 (2-16)	-85.3% [-98.0 a -72.7]	<0.001
Look-downs per minute	0 (0-1.0)	1.6 (1.0-7.0)	-78.4% [-96.2 a -60.6]	<0.001
Number of interventions	1.0 (0-2.0)	4.5 (1.0-10.0)	-75.9% [-88.7 a -63.1]	<0.001
Interventions per minute	0.5 (0-1.0)	2.0 (0.5-4.0)	-60.2% [-83.8 a -36.6]	<0.001
Number of instrument loss episodes	0 (0-2)	3 (2-10)	-92.8% [-100.3 a -85.3]	<0.001
Loss episodes per minute	0 (0-2)	1.3 (1.0-2.5)	-88.2% [-99.9 a -76.5]	<0.001
Total loss duration, s	0 (0-6)	10 (6-55)	-93.3% [-100.6 a -86.0]	<0.001
% of total loss duration	0 (0-7)	8 (4-23)	-91.2% [-100.4 a -82.1]	<0.001

Data are described as median (range) and compared using the Wilcoxon test.*Relative difference (%) = ((value in the intervention group - value in the control group) / value in the control group) × 100. [95% CI]: 95% confidence interval.

The total GOALS score was significantly higher for the intervention group than the control group. Simulator-trained participants also scored significantly higher in all GOALS components (depth perception, bimanual dexterity, efficiency, tissue handling, and autonomy) (Table 2).

**Table 2:** Global Operative Assessment of Laparoscopic Skills (GOALS) scores in the intervention (simulator training) and control (no simulator training) groups.

GOALS score	Interventionn=20	Control n=20	p	r*
Total	23 (17-25)	8 (5-15)	<0.001	0.88
Depth perception	5 (3-5)	1 (1-3)	<0.001	0.92
Bimanual dexterity	5 (3-5)	1 (1-3)	<0.001	0.92
Efficiency	5 (3-5)	2 (1-3)	<0.001	0.89
Tissue handling	5 (3-5)	3 (1-3)	<0.001	0.84
Autonomy	5 (3-5)	1 (1-3)	<0.001	0.92

Data are described as median (range) and compared using the Wilcoxon test. *Effect size (r): r<0.30, small; 0.30<r<0.50, medium; r>0.50, large.

In the experience questionnaire, all participants assigned maximum scores to statements concerning the utility of using a simulator and its contribution to learning. However, most students indicated that they did not believe simulator training could replace actual surgical training (Table 3).

**Table 3:** Student experience.

Question*	Intervention n=20	Control n=20	p	r*
Do you believe a spine simulator could enhance learning in this course?	5 (4-5)	5 (4-5)	0,102	0,37
Would you like simulator training to be integrated into your undergraduate program?	5 (4-5)	5 (4-5)	0,137	0,22
Do you believe simulator training could replace actual surgical training?	2 (1-4)	2 (1-5)	0,396	0,19
Do you find the instructional video sufficient preparation for performing the exercise in real surgery?	3 (1-5)	2.5 (1-5)	0,719	0,08

Data are described as median (range) and compared using the Wilcoxon test.*Responses range from 1 (strongly disagree) to 5 (strongly agree).†Effect size (r): r<0.30, small; 0.30<r<0.50, medium; r>0.50, large.

## DISCUSSION

Surgical simulation is increasingly regarded as an essential tool for teaching technical skills, especially in minimally invasive procedures. This study is the first to demonstrate transfer validity in spinal endoscopy, highlighting the impact of prior training in an accessible synthetic simulator on performance in a real surgical setting. The proposed model contributes to the advancement of education in endoscopic spine surgery by providing a viable alternative for initial training, using a controlled and measurable methodological approach^9^ ^-^ ^14^.

The selection of medical students as the primary study sample was a strategic methodological decision. This group provides greater homogeneity due to their equivalent initial surgical skills, a crucial factor for studies involving randomized allocation. Surgical residents, even when matched for training level, often demonstrate wide technical variability, which may compromise the validity of comparisons. Specialist surgeons, in turn, already master the target skills, precluding the analysis of the impact of simulated training. Furthermore, recruiting the large sample sizes necessary for adequate statistical power is only feasible among students, given the limited availability of novice residents for simultaneous randomization. Therefore, using medical students allowed for enhanced experimental control and reinforces the applicability of the simulator to the initial stages of medical training, consistent with previous construct validity studies^5^ ^,^ ^15^ ^,^ ^16^.

Simulator validation has relied on indirect methods, including face, content, and construct validity. However, the literature lacks evidence regarding transfer validity, which is crucial for demonstrating the efficacy of simulated training in real-world clinical practice, especially in the context of lumbar spinal endoscopy^14^ ^-^ ^16^. This study fills this gap by objectively demonstrating the transfer of skills from the simulation setting to the operating room, providing novel evidence in the field.

The use of the simulator resulted in an 80% improvement in overall performance, with substantial reductions in both procedure time and the need for supervisor involvement. Simulator-trained participants showed better technical fluidity and autonomy, even without longitudinal assessment of multiple procedures. These data reinforce that simulation-based training can anticipate stages of the learning curve and facilitate the transition to endoscopic practice.

Simulator training significantly improved several operative parameters. Notably, simulator-trained participants demonstrated a 43.7% reduction in the total duration of real surgical procedures. Operative time is widely recognized as a key indicator of technical efficiency^5^ ^,^ ^16^ ^,^ ^17^, mainly because of its role in minimizing complications, particularly surgical site infections. Previous studies indicate that simulators can reduce operative time by up to 30%^18^. The substantial reduction in total procedure time observed in this study may be related to the participants’ lack of prior experience with spinal endoscopy, highlighting the importance of this type of simulator in the initial stages of technical skills training.

This study also demonstrated a significant reduction in variables related to hand-eye coordination in simulator-trained participants. A key finding was the 85.3% reduction in the number of look-downs. This metric is particularly relevant for procedures requiring indirect visualization, such as spinal endoscopy, in which spatial orientation depends on the surgeon’s ability to interpret a two-dimensional image. Previous studies have used this indicator as a measure of spatial orientation in laparoscopic simulation, demonstrating improvements of up to 60%^16^ ^,^ ^19^. The more substantial reduction observed in our study may be attributed to the use of the simulator as a pre-operative warm-up, a strategy associated with neuromotor activation and improved operative fluency^20^.

Other findings of this study further support the efficacy of simulation in surgical training. Instrument loss was reduced in 92.8% of cases, total duration of instrument loss in 93.3%, and interventions by the supervising surgeon in 75.9%. Previous studies using simulators have shown reductions of 50% to 70% in these same indicators^21^ ^-^ ^23^. Our results are consistent with these findings and indicate considerable objective improvements with simulator training, especially because these gains were achieved in a single supervised practice session.

The assessment of surgical performance was complemented by the GOALS scores^5^ ^,^ ^24^. Simulator-trained participants scored three times higher in all GOALS components than control participants. To date, no published studies have used GOALS scores in transfer validity for spinal endoscopy simulators. While systematic reviews, such as that by Morgan et al.^25^, have reported improvements of up to 26% using simulators, these studies have not evaluated the transfer of skills to the operating room and have included participants with different levels of experience, which can compromise the homogeneity of the sample and limit the interpretation of the results. The combination of transfer validity and a homogeneous sample, as adopted in this study, increases methodological rigor and reinforces the applicability of the GOALS rating scale for assessing technical skills in the context of spinal endoscopy.

Our subjective evaluation, using a Likert scale, revealed a high level of acceptance for the simulator. All participants expressed interest in incorporating simulator training into the undergraduate medical curriculum, suggesting that simulated practice generated substantial engagement^26^. Analysis of the previously provided instructional video indicated limited utility of this resource in developing technical skills, underscoring the role of active simulation as a key element for the successful acquisition of skills^27^ ^,^ ^28^.

The model developed in this study is a low-fidelity simulator^28^, which, despite its structural simplicity, can replicate essential technical maneuvers of spinal endoscopy, including triangulation and hand-eye coordination. While challenges were encountered in the simulation of soft tissues, the use of foam to fill the manikin and EVA sheets to represent the ligamentum flavum proved to be effective strategies^5^ ^,^ ^10^. The model’s low cost and ease of use render it suitable for implementation across different learning stages, from undergraduate programs to the first years of residency.

Some countries still lack formal requirements for simulation-based training prior to patient-based training in residency programs. Several factors hinder the implementation of simulation, such as the absence of standardization, production costs, and the need for model validation. Conversely, countries such as the United States and France have already adopted mandatory simulation training protocols^28^ ^-^ ^30^. Our findings provide concrete evidence to support a gradual shift in this paradigm.

The brief ten-minute simulator warm-up performed before the actual surgery may have contributed to the positive results, which is consistent with previous studies reporting an association between pre-operative warm-up and improved technical performance^19^ ^,^ ^20^. This suggests that the incorporation of a warm-up routine could be a valuable strategy for optimizing performance, especially in the initial stages of surgical training. Furthermore, the analysis by an independent, blinded examiner increases the reliability of our findings and mitigates the risk of detection bias^31^.

Our study has limitations that should be acknowledged. The simulator was not designed to include training for the initial stages of the surgical approach that involve radioscopy, thus limiting its scope. The sample consisted solely of medical students from a single institution, which may limit the generalizability of the results. The incorporation of actual instruments into the model increased its cost; however, the functionality of the simulator remained viable. Moreover, retention of the acquired skills was not assessed over time. Future studies should include a more diverse sample, compare the simulator with other models, and assess long-term retention of learned skills.

## CONCLUSION

The synthetic spinal endoscopy simulator demonstrated, in a novel and methodologically controlled manner, transfer validity by significantly improving all technical performance indicators in real surgery. Furthermore, GOALS scores tripled among simulator-trained participants, and the simulator was accepted by 100% of participants as applicable to medical education.

## References

[1] Ruetten S, Komp M, Merk H, Godolias G (2008). Full-endoscopic interlaminar and transforaminal lumbar discectomy versus conventional microsurgical technique a prospective, randomized, controlled study. Spine (Phila Pa 1976).

[2] Bonafim RMS, Kulcheski AL, Sebben AL, Del Santoro PG, Benato ML, I-Graells XS (2023). Interlaminar endoscopic lumbar discectomy - clinical outcome. Coluna/Columna.

[3] Ransom NA, Gollogly S, Lewandrowski KU, Yeung A (2020). Navigating the learning curve of spinal endoscopy as an established traditionally trained spine surgeon. J Spine Surg.

[4] Sebben AL, Kulcheski ÁL, Graells XSI, Benato ML, Santoro PGD (2021). Comparison of two endoscopic spine surgical techniques. Rev Assoc Med Bras (1992).

[5] Kulcheski ÁL, Stieven-Filho E, Nunes CP, Milcent PAA, Dau L, I-Graells XS (2021). Validation of an endoscopic flavectomy training model. Rev Col Bras Cir.

[6] Bohm PE, Arnold PM (2015). Simulation and resident education in spinal neurosurgery. Surg Neurol Int.

[7] Van Nortwick SS, Lendvay TS, Jensen AR, Wright AS, Horvath KD, Kim S (2010). Methodologies for establishing validity in surgical simulation studies. Surgery.

[8] Dawe SR, Pena GN, Windsor JA, Broeders JA, Cregan PC, Hewett PJ (2014). Systematic review of skills transfer after surgical simulation-based training. Br J Surg.

[9] Lohre R, Wang JC, Lewandrowski KU, Goel DP (2020). Virtual reality in spinal endoscopy a paradigm shift in education to support spine surgeons. J Spine Surg.

[10] Nunes CP, Kulcheski AL, Almeida PA, S E, Graells XS (2020). Creation of a low-cost endoscopic flavectomy training model. Coluna/Columna.

[11] Likert R (1932). A technique for the measurement of attitudes. Arch Psychol.

[12] Abramson JH (2011). WINPEPI updated computer programs for epidemiologists, and their teaching potential. Epidemiol Perspect Innov.

[13] Ross JAG, Sampson N, Martins DE, Astur N (2024). Perception of the learning curve for endoscopic spine procedures, a survey of spinal surgeons in LATAM. Coluna/Columna.

[14] Satava RM (2007). The future of surgical simulation and surgical robotics. Bull Am Coll Surg.

[15] McDougall EM (2007). Validation of surgical simulators. J Endourol.

[16] Dau L, Almeida PA, Milcent PAA, Rosa FM, Kulcheski AL, Stieven E (2021). Shoulder arthroscopy - creating an affordable training model. Rev Bras Ortop (Sao Paulo).

[17] Alvand A, Khan T, Al-Ali S, Jackson WF, Price AJ, Rees JL (2012). Simple visual parameters for objective assessment of arthroscopic skill. J Bone Joint Surg Am.

[18] Coelho G, Vieira T (2018). História da simulação cirúrgica e sua aplicação em Neurocirurgia. Sci Med.

[19] Chen CC, Green IC, Colbert-Getz JM (2013). Warm-up on a simulator improves residents' performance in laparoscopic surgery a randomized trial. Int Urogynecol J.

[20] Plerhoples TA, Zak Y, Hernandez-Boussard T, Lau J (2011). Another use of the mobile device warm-up for laparoscopic surgery. J Surg Res.

[21] Ghobrial GM, Hamade YJ, Bendok BR, Harrop JS (2015). Technology and simulation to improve patient safety. Neurosurg Clin N Am.

[22] Liu JK, Page PS, Brooks NP (2021). Development and validation of a low-cost endoscopic spine surgery simulator. Cureus.

[23] Shahrezaei A, Sohani M, Taherkhani S, Zarghami SY (2024). The impact of surgical simulation and training technologies on general surgery education. BMC Med Educ.

[24] Vassiliou MC, Feldman LS, Andrew CG (2005). A global assessment tool for evaluation of intraoperative laparoscopic skills. Am J Surg.

[25] Morgan M, Aydin A, Salih A, Robati S, Ahmed K (2017). Current status of simulation-based training tools in orthopedic surgery a systematic review. J Surg Educ.

[26] Harrop J, Rezai AR, Hoh DJ, Ghobrial GM, Sharan A (2013). Neurosurgical training with a novel cervical spine simulator posterior foraminotomy and laminectomy. Neurosurgery.

[27] Moura-Júnior LG, Ramos A, Campos JM, Ferraz ÁA, Rocha HÂL, Costa GO (2017). Teaching model for evaluation of the ability and competence progress in endosuture in surgical skill laboratory. Arq Bras Cir Dig.

[28] Atesok K, Hurwitz S, Anderson DD (2019). Advancing simulation-based orthopaedic surgical skills training an analysis of the challenges to implementation. Adv Orthop.

[29] Cunha CMQD, Lima DMF, Menezes FJC (2018). Low-cost simulator assembly for 3-dimensional videosurgery training. Arq Bras Cir Dig.

[30] Seil R, Hoeltgen C, Thomazeau H, Anetzberger H, Becker R (2022). Surgical simulation training should become a mandatory part of orthopaedic education. J Exp Orthop.

[31] Malavolta EA, Demange MK, Gobbi RG, Imamura M, Fregni F (2015). Randomized controlled clinical trials in orthopedics difficulties and limitations. Rev Bras Ortop.

